# Dynamics of gene expression associated with arsenic uptake and transport in rice during the whole growth period

**DOI:** 10.1186/s12870-020-02343-1

**Published:** 2020-03-31

**Authors:** Dandan Pan, Jicai Yi, Fangbai Li, Xiaomin Li, Chuanping Liu, Weijian Wu, Tingting Tao

**Affiliations:** 1 SCNU Environmental Research Institute, Guangdong Provincial Key Laboratory of Chemical Pollution and Environmental Safety & MOE Key Laboratory of Theoretical Chemistry of Environment, South China Normal University, Guangzhou, 510006 China; 2 Guangdong Institute of Eco-Environmental Science & Technology, Guangdong Key Laboratory of Integrated Agro-environmental Pollution Control and Management, Guangzhou, 510650 China; 3 College of Natural Resources and Environment, South China Agricultural University, Guangzhou, 510642 China; 4 School of Environment, South China Normal University, Guangzhou, 510006 China; 5 College of Life Sciences, South China Agricultural University, Guangzhou, 510642 China; 6 School of Food Science and Engineering, Foshan University, Foshan, 528000 China

**Keywords:** Arsenite, Uptake and transport, Gene expression, Rice, Whole growth period

## Abstract

**Background:**

Genes associated with arsenite uptake and transport in rice plants (i.e., *OsLsi1*, *OsLsi2*, *OsLsi3*, *OsLsi6* and *OsABCC1*) have been identified to date. However, their expression over time during the whole growth period of rice under arsenite stress conditions is still poorly understood. In this study, the dynamics of gene expression associated with arsenite transport and arsenic concentrations in different organs of rice were investigated to determine the critical period(s) of arsenite uptake and translocation regulated by gene expression during the whole growth period.

**Results:**

The relative expression of *OsLsi2* and *OsLsi1* in the roots was upregulated and reached its highest value (2^-∆∆Ct^ = 4.04 and 1.19, respectively) at the jointing stage (9 weeks after transplantation), in which the arsenic concentration in roots also was the highest at 144 mg/kg. A range from 45.1 to 61.2% of total arsenic accumulated in the roots during seedling to heading stages (3–16 weeks), which was mainly associated with the relatively high expression of *OsABCC1* (1.50–7.68), resulting in arsenic located in the vacuoles of roots. Subsequently, the As translocation factor from root to shoot increased over time from heading to milky ripe (16–20 weeks), and 74.3% of the arsenic accumulated in shoots at the milk stage. Such an increase in arsenic accumulation in shoots was likely related to the findings that (i) *OsABCC1* expression in roots was suppressed to 0.14–0.75 in 18–20 weeks; (ii) *OsLsi3* and *OsABCC1* expression in nodes I, II, and III was upregulated to 4.01–25.8 and 1.59–2.36, respectively, in 16–20 weeks; and (iii) *OsLsi6* and *OsABCC1* expression in leaves and husks was significantly upregulated to 2.03–5.26 at 18 weeks.

**Conclusions:**

The jointing stage is the key period for the expression of arsenite-transporting genes in roots, and the heading to milky ripe stages are the key period for the expression of arsenite-transporting genes in shoots, both of which should be considered for regulation during safe rice production in arsenic-contaminated paddy soil.

**Graphical abstract:**

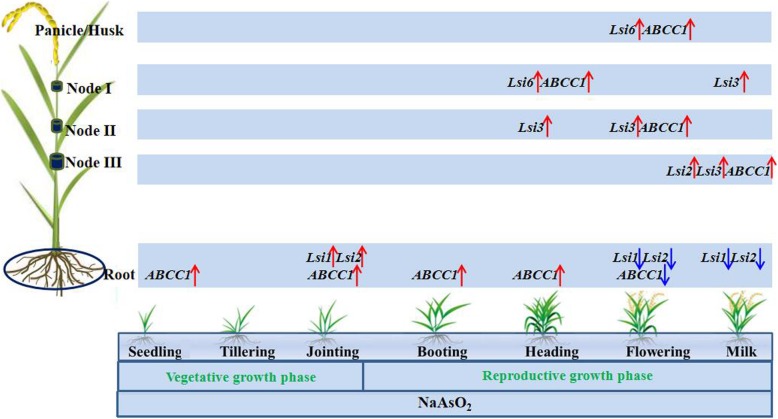

## Background

Arsenic (As) contamination in soils and water has become a serious environmental problem, especially in South and Southeast Asia [1, 2]. Mining and industrial activities are the main sources of As contamination to the environments [3–5]. The As content in rice grains produced from contaminated sites in China, India and Korea can be as high as 0.77–0.85 mg/kg [6, 7]. In paddy soil, arsenite (As(III), H_3_AsO_3_) is the predominant species of As that is taken up by rice [8–10]. Understanding the regulations and key period of As(III) uptake and transport by rice is important to developing control strategies for safe rice production in As-contaminated soils.

To date, genes that have been identified to be associated with As(III) uptake and transport in rice plants are the same as those for silicon (Si) uptake and transport because arsenite is a chemical analogue of silicic acid. In rice roots, As(III) is inadvertently taken up and transported via the silicic acid transporters OsLsi1 and OsLsi2 [8, 11, 12]. OsLsi1 is preferentially distributed on the distal side of Casparian bands, passively transporting As(III) into root cells; OsLsi2, localized on the proximal side of Casparian bands, actively transports As(III) from root cells to apoplast toward xylem [8, 13, 14]. Once transported into the root cells, As(III) can be either complexed with phytochelatins (PCs) and then sequestered in vacuoles for detoxification [15, 16] or transported to stems and leaves by transpirational flow through the xylem vessels of rice [17]. OsABCC1, a C-type ABC (ATP-binding cassette) transporter localized in the tonoplast, is responsible for As vacuolar compartmentalization [16]. *OsABCC1* can be expressed in roots, stems, leaves and husks of rice, and sequestering As in vacuoles is important in reducing the allocation of As to rice grains [16].

Nodes in graminaceous plants control the distribution of mineral elements in different tissues of shoots, including essential and toxic elements [18, 19]. In the nodes of rice, three transporters (i.e., OsLsi6, OsLsi2, and OsLsi3) are involved in the intervascular transport of As(III) from nodes to panicles [20–22]. OsLsi6, a plasma membrane-localized Si/As(III) channel, is mainly expressed at the xylem transfer cells of enlarged vascular bundles (EVBs) [23]. OsLsi2 and OsLsi3 are localized at the distal side of the bundle sheath of EVBs and parenchyma cells between EVBs and diffuse vascular bundles (DVBs), respectively [22]. As(III) in the xylem of EVBs can be selectively unloaded by OsLsi6 and then reloaded to the xylem of DVBs by OsLsi2 and OsLsi3, leading to preferential As distribution to panicles through xylem vessels [22, 23]. In addition, OsLsi6 can be found in leaves and nodes and is responsible for As(III) transport out of xylem into the tissues of leaf and node [8].

A number of studies have explored As(III) uptake and transport in rice plants [19, 22, 24], the majority of which mainly focused on the seedling or maturing stage of rice growth [25–27]. For example, many experiments implemented to identify the As(III) transporters in rice (e.g., OsLsi1 and OsLsi2) were performed during the seedling stage [25–27]. Arsenic is mainly transported into caryopsis during the grain filling stage [28], which is considered to be the key stage to take measures to reduce As uptake in rice [29]. However, As(III) can be taken up by rice during the whole growth period, and As(III) transport in different organs and/or tissues of rice is mediated by various transporters, as mentioned above. The expression of genes for these transporters is important to regulating As accumulation in grains. However, gaps in our understanding remain with regard to the dynamics in gene expression of As(III) uptake and transport during the whole growth period. Thus, the aim of the present study was to investigate the dynamics of gene expression of As(III)-related transporters as well as the characteristics of As(III) uptake and accumulation in different organs of rice during the whole growth period. The results obtained can provide a better understanding of the As(III) uptake and transport regulated by gene expression in different parts of rice, which would be useful to guide As mitigation strategies in As-contaminated paddy soil.

## Results

### Arsenic distribution in rice plants during the whole growth period

**Fig. 1:**
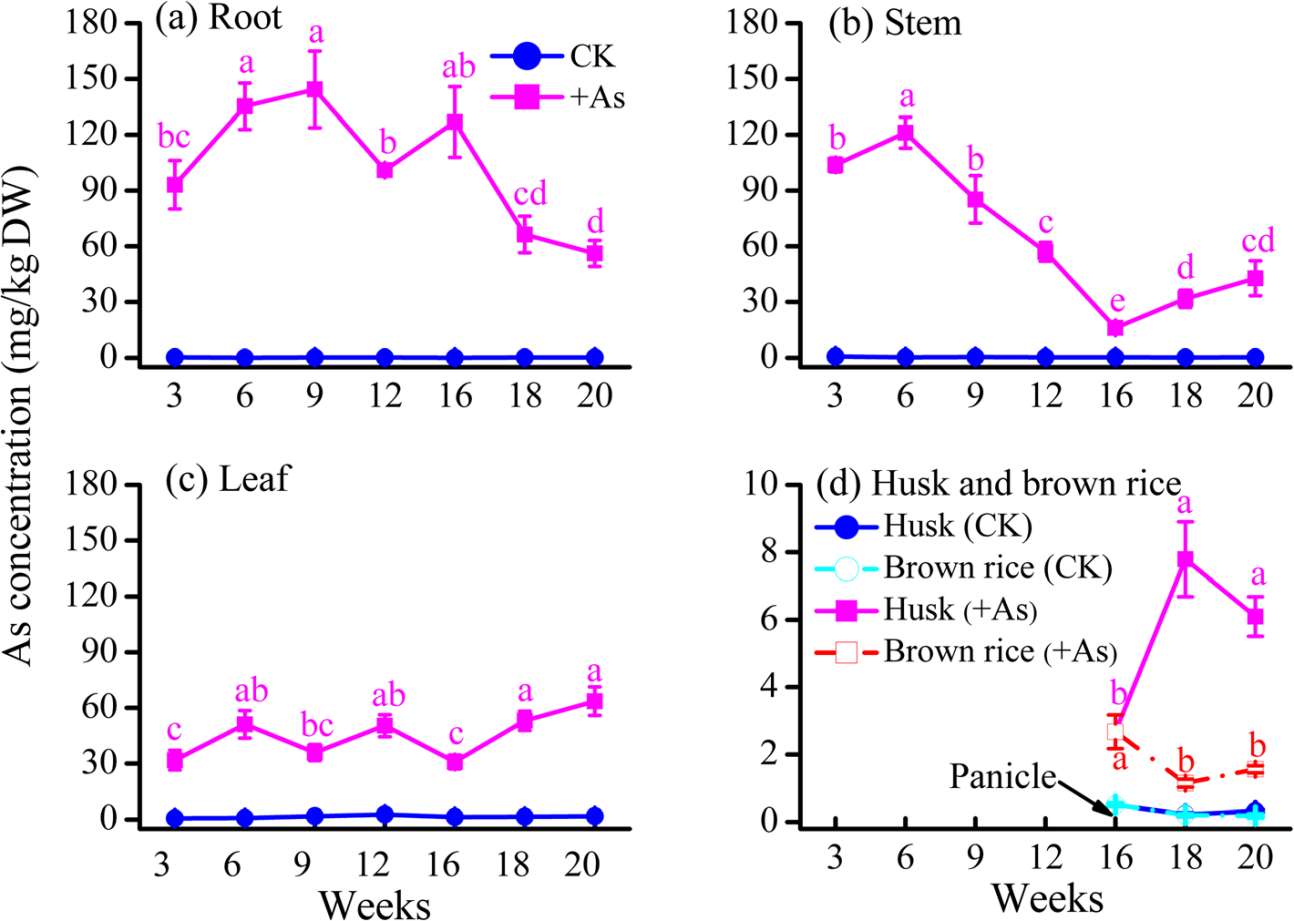
The As concentration in different organs of rice plants during the whole growth period. **a** Root, **b** stem, **c** leaf, **d** husk and brown rice. Plants were grown under hydroponic conditions with 5 μM NaAsO_2_ (+As treatment) or without it (the control, CK). Data are presented as the mean ± SE (*n* = 3). Different letters with colors corresponding to their respective lines indicate significant differences (*P* < 0.05) among values at different time intervals in the +As treatment

Generally, the total As concentrations in different organs of rice ranked in the following order: root > stem ≥ leaf > husk > brown rice in the +As treatment (Fig. 1). The As concentration in roots increased over time from the seedling to jointing stages (3–9 weeks) and reached its highest value of 144 mg/kg at the jointing stage (9 weeks) (Fig. 1a). During heading to milk stages (16–20 weeks), the As concentration in roots decreased from 126 mg/kg to 56.1 mg/kg, while those in stems and leaves increased from 16.1 mg/kg to 42.7 mg/kg and from 30.9 mg/kg to 63.6 mg/kg, respectively (Fig. 1a-1c). At the same time, the As concentrations in husks were higher than those in brown rice during the heading to milk stages (Fig. 1d). The results in Figure S1 show that the majority of the biomass of roots, stems, leaves and grains in the +As treatment were similar to those in the CK treatment, except for the decrease in root and leaf biomass at 12 weeks (*P* < 0.05) and decrease in grain yields at 20 weeks (*P* < 0.05).

**Fig. 2:**
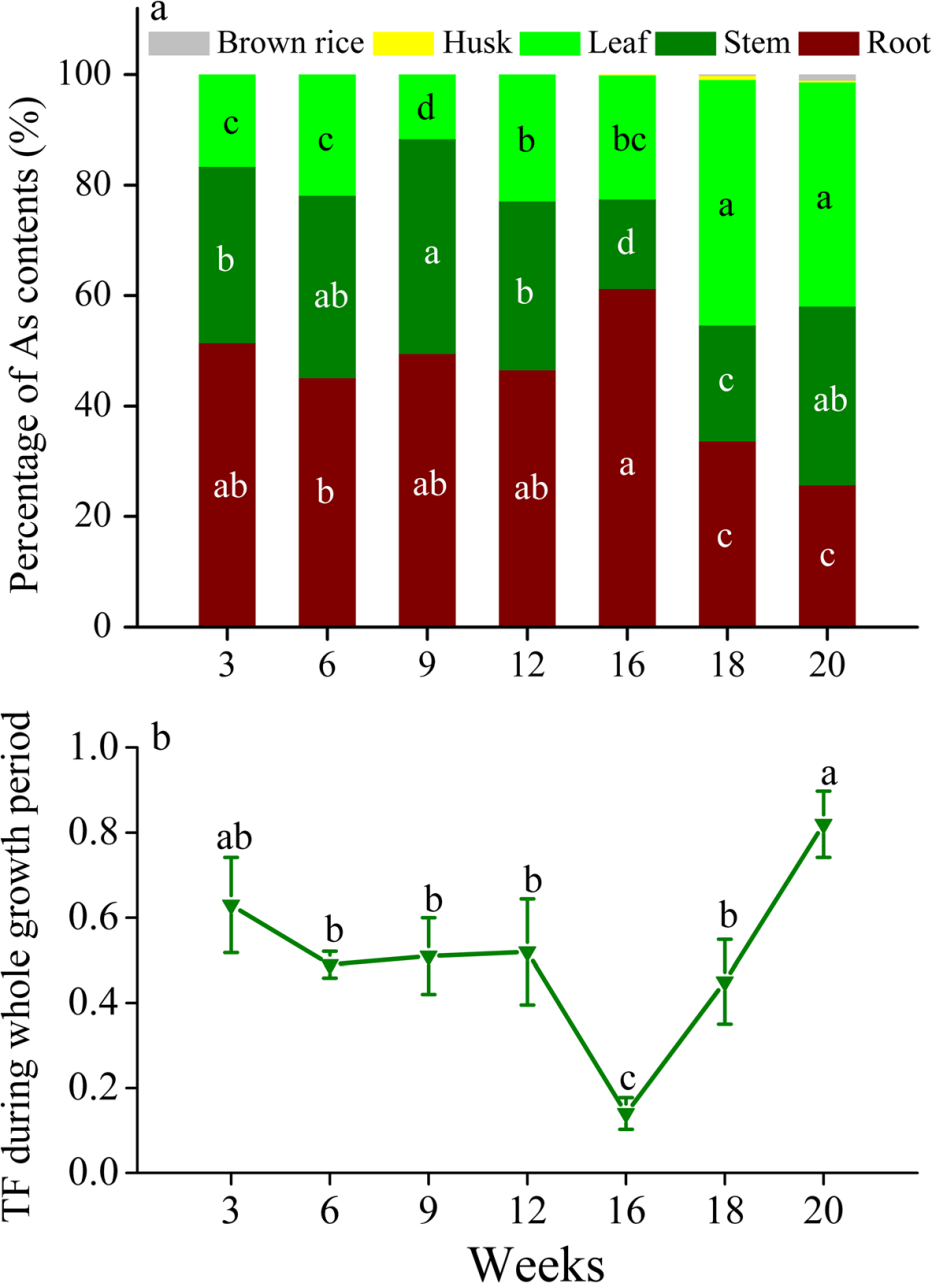
**a** Percentage of As content in the different organs of rice plants and **b** translocation factor (TF) during the whole growth period of the +As treatment. Columns and line labeled by different letters are significantly different at a *P* < 0.05 level among values at different time intervals

In the +As treatment, the total As content in roots was the highest at 938 μg at the heading stage (16 weeks), while those in stems and leaves were the highest at 889 μg and 1116 μg at the milk stage, respectively (Figure S2a). During the seedling to heading stages (3–16 weeks), 45.1–61.2% of the As taken up by rice accumulated in roots, while 66.4–74.3% accumulated in shoots (particularly 40.6–44.4% in leaves) during the flowering to milk stages (Fig. 2a). The translocation factor (TF) remained stable at a range of 0.49–0.63 before the booting stage (12 weeks), then decreased to a minimum value of 0.14 at the heading stage (16 weeks), and then increased linearly over time to a maximum value of 0.82 at the milk stage (20 weeks) (Fig. 2b). These results indicate that the seedling to heading stages are the period for As uptake and accumulation in roots, while the heading to milk stages are the period for As transport from roots to shoots during the whole growth period of rice.

### Expression of arsenite-related genes in roots

**Fig. 3:**
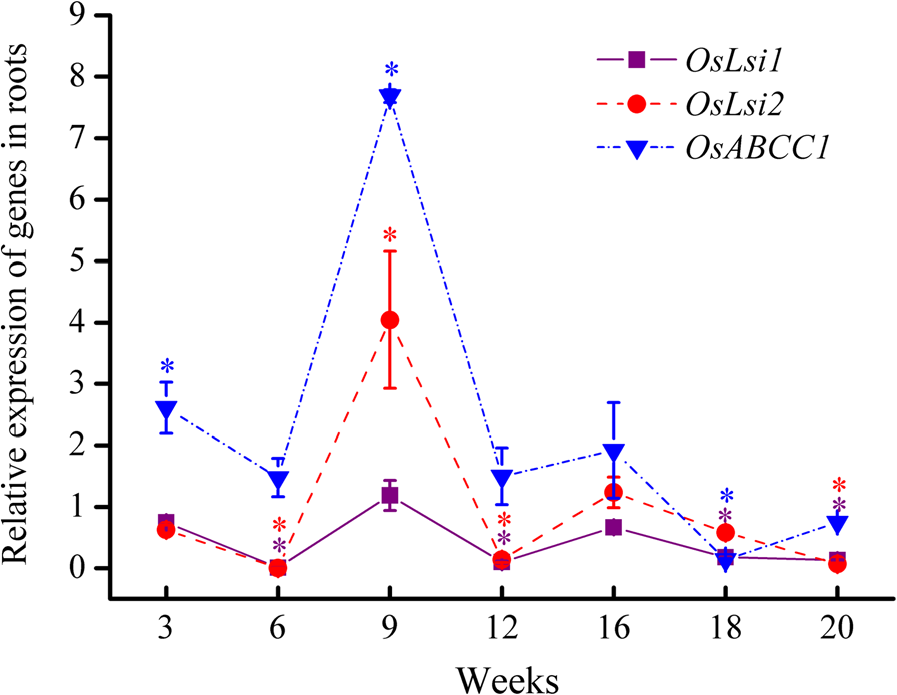
Relative expression of *OsLsi1*, *OsLsi2* and *OsABCC1* genes in roots of rice in the +As treatment relative to those in the control treatment during the whole growth period. Data are presented as the mean ± SE (*n* = 3). Lines labeled by * with colors corresponding to their respective lines are significantly different at a *P* < 0.05 level in comparison to the control treatment

The results in Fig. 3 show that the *OsLsi1* and *OsLsi2* genes are constitutively expressed in roots during the whole growth period of the +As treatment, which is consistent with previously reported results [14, 26]. In this study, their relative expression varied with time. While the relative expression of the *OsLsi1* and *OsLsi2* genes was significantly suppressed to < 0.13 at 6 and 12 weeks (*P* < 0.05), their expression was disinhibited or promoted to 1.19 and 4.04 at 9 weeks and to 0.67 and 1.23 at 16 weeks, respectively, followed by a decline over time after 16 weeks.

The relative expression of the *OsABCC1* gene in roots was obviously promoted (≥ 1.48 during the seedling to heading stages and had the maximum value of 7.68 at the jointing stage (9 weeks), whereas it was suppressed to below 0.75 after the heading stage. These results suggested that As sequestering in vacuoles of roots is active during the period of arsenite uptake and accumulation in roots (seedling to heading stages). In addition, the relative expression of *OsABCC1* in roots was linearly positively related to those of *OsLsi1* and *OsLsi2* in roots (Figure S3), which indicated that if more As(III) uptake occurs in roots, then more As(III) is accumulated in the vacuoles of roots.

### Expression of arsenite-related genes in shoots

**Fig. 4:**
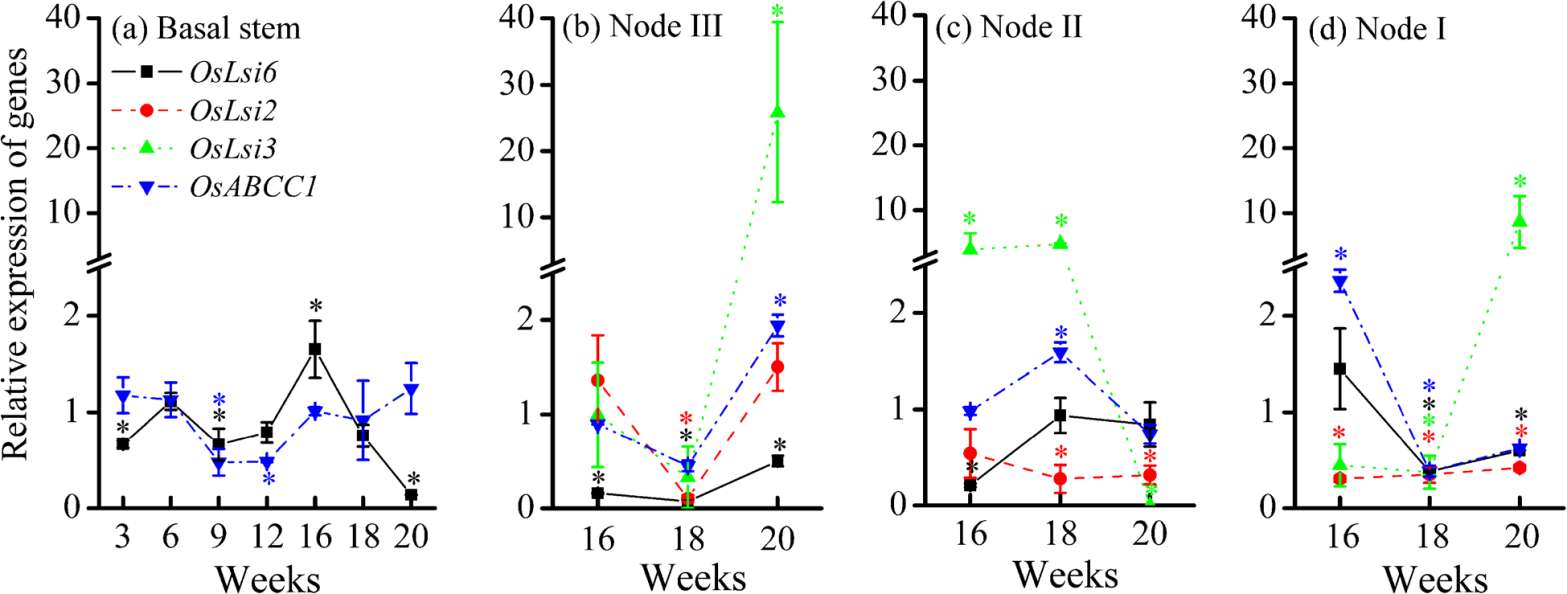
Relative expression of *OsLsi6*, *OsLsi2*, *OsLsi3* and *OsABCC1* genes in (**a**) basal stem, (**b**) node III, (**c**) node II, and (**d**) node I of rice in the +As treatment relative to those in the control treatment during the whole growth period. Data are presented as the mean ± SE (*n* = 3). Lines labeled by * with colors corresponding to their respective lines are significantly different at a *P* < 0.05 level in comparison to the control treatment

In the basal stems, the relative expression of *OsABCC1* was maintained at a range of 0.48–1.25 during the whole growth period, while that of *OsLsi6* increased from 0.66 at the seedling stage to the highest value of 1.65 at the heading stage and then significantly decreased to 0.14 (*P* < 0.05) at the milk stage (Fig. 4a). Such an inhibition of gene expression of *OsLsi6* in the basal stems at 20 weeks could result in more As accumulated in the unelongated stems and more As transported to the bottom leaves, which are connected to the unelongated nodes in the basal stem via xylem vessels.

In nodes III, II, and I, the relative expression of *OsLsi3* and *OsABCC1* could be upregulated to 4.01–25.8 and 1.59–2.36, respectively, for 16–20 weeks, while the majority of *OsLsi6* relative expression was < 1.0 and those of *OsLsi2* were only promoted to 1.36–1.50 in node III (Fig. 4b-d). On the other hand, the expression levels of *OsABCC1*, *OsLsi6*, *OsLsi3*, and *OsLsi2* were also suppressed (< 1.0) sometimes, particularly in nodes III and I at 18 weeks (Fig. 4b and d). The inhibition of the gene expression of *OsLsi6*, *OsLsi3*, and *OsLsi2* at 18 weeks in node I could retain more As accumulated in node I or transport it to the top first leaf, while the promotion of their gene expression in the nodes may lead to more As transported to grains.

**Fig. 5:**
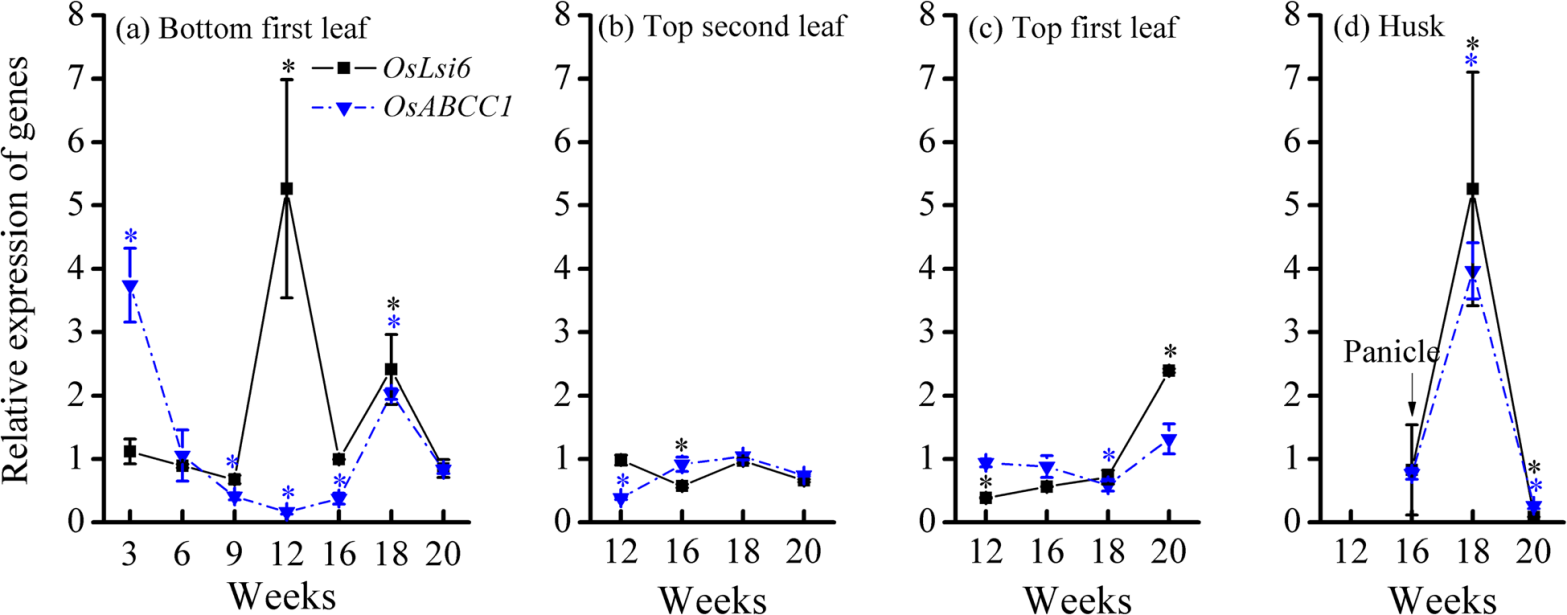
Relative expression of *OsLsi6* and *OsABCC1* genes in (**a**) bottom first leaf, (**b**) top second leaf, (**c**) top first leaf, and (**d**) husk of rice in the +As treatment relative to those in the control treatment during whole growth period. Data are presented as the mean ± SE (*n* = 3). Lines labeled by * with colors corresponding to their respective lines are significantly different at a *P* < 0.05 level in comparison to the control treatment

For the bottom first leaf, significant upregulation of gene expression (*P* < 0.05) was observed for the *OsLsi6* gene at 12 and 18 weeks and for the *OsABCC1* gene at 3 and 18 weeks (Fig. 5a). For the top leaves, the relative expression of *OsLsi6* and *OsABCC1* was only observed to be upregulated in the top first leaf at 20 weeks, while the remainder was maintained at a range of 0.38–1.0 for 12–20 weeks (Fig. 5b and c). In the husks, the relative expression of *OsLsi6* and *OsABCC1* was significantly promoted to 5.26 and 3.97 at 18 weeks, respectively (*P* < 0.05) and then suppressed to < 0.54 at 20 weeks (Fig. 5d). Hence, the gene expression of *OsLsi6* and *OsABCC1* was mainly upregulated in the bottom first leaf and husks but not in the flag leaves (i.e., top first and second leaves) from the booting to milk stages.

Since arsenite is generally complexed with PCs before being sequestered in the vacuoles for detoxification [16], and the biosynthesis of PCs is catalyzed by the phytochelatin synthase such as PCS1 [30], the expression of *OsPCS1* gene in different organs of rice was quantified as well. As shown in Figure S4-S6, the *OsPCS1* and *OsABCC1* genes are constitutively expressed in roots, nodes, and husks in the +As treatment, which confirmed that vacuolar sequestration of As is strongly associated with its complexation by PCs.

## Discussion

Generally, the whole growth period of rice plants includes seven growth stages, i.e., seedling, tillering, jointing, booting, heading, flowering, and milk stages, which can be categorized into vegetative growth or reproductive growth phases [29]. It is difficult to identify the time of transition from the vegetative growth to reproductive growth phases (i.e., the initiation of panicle differentiation), and both vegetative growth and reproductive growth phases are usually believed to proceed simultaneously during the jointing to heading stages [29, 31]. Nonetheless, the duration of the vegetative growth phase in rice is considered from the seedling to jointing stages (3–9 weeks) and that of the reproductive growth phase from the booting until milk stages (12–20 weeks) in this study.

### Key gene expression in the vegetative growth phase

The vegetative growth phase is an important period for the formation of the root system of rice [29], which is the first organ to absorb arsenite and to respond to the toxic effect of arsenite accumulated in rice [32]. In the vegetative growth phase of this study, it was obvious that As concentrations in roots were higher than those in stems and leaves (Fig. 1), and the majority of As was accumulated in roots, with the total As content accounting for 45.1–51.4% (Fig. 2a). During this phase, we observed an inhibition of the gene expression of *OsLsi1* and *OsLsi2* in roots at the tillering stage (Fig. 3), which is consistent with previous results of field experiments [26, 33]. Such an inhibition is probably due to a self-protecting response of rice to toxic As [34], which could be one of the reasons for the decrease in the percentage of As content in roots at this stage (Fig. 2a). Subsequently, the disinhibition of *OsLsi1* and promotion of *OsLsi2* expression in roots at the jointing stage (Fig. 3) was attributed to a demand for Si for rice growth and/or because the transporters OsLsi1 and OsLsi2 were degraded and needed to be recovered [26, 33]. Such a promotion of *OsLsi1* and *OsLsi2* expression can result in an increase in arsenite uptake by roots (Fig. 1 and S2a).

Once arsenite is taken up by roots, vacuolar sequestration of As by the transporter OsABCC1 can limit the mobility of arsenite and control the transfer of arsenite to other organs in plants [16, 35]. *OsABCC1* expression in the bottom first leaf was highest at the seedling stage (Fig. 5a), which may result in a preferential accumulation of As in the vacuoles of bottom leaves. At the jointing stage, notably, *OsABCC1* expression in roots showed the highest value (Fig. 3), while this expression was suppressed in the basal stem and bottom first leaf (Fig. 4 and 5a). Such a difference in *OsABCC1* expression between roots and shoots not only led to the increase in As concentration in roots but also resulted in the decrease in As concentration in shoots (Fig. 1). The decrease in the TF value from the seedling to tillering/jointing stages (Fig. 2b) confirms that although the total As contents in the roots, stems and leaves increased gradually as their biomass grew (Figure S1 and S2), less As was transferred from the roots to the shoots during the vegetative growth phase. As a result, the promotion of *OsABCC1* expression in roots is the key regulation to sustaining high As accumulation in roots and to restraining As transfer from roots to shoots during the vegetative growth phase.

During the vegetative phase, the expression patterns of *OsLsi1*, *OsLsi2* and *OsABCC1* in roots in this study were different from those in previous studies [16, 26, 33], which might be due to the fact that the nutrient solution of this study was Si-free, and the paddy soils in their field experiments contained Si. While these transporters are predominantly responsible for As(III) uptake in this study, the presence of Si may prevail against As(III) since Si and As(III) use the same transporters for uptake by rice as that in the previous studies. High Si accumulation in shoots can decrease As uptake and accumulation through downregulation of the expression of *OsLsi1* and *OsLsi2* in roots [36]. Si fertilizer has been demonstrated to hinder *OsLsi1* and *OsLsi2* expression in roots [26, 33], and it can be applied to rice plants grown in arsenic-contaminated paddy soils at the jointing stage.

### Key gene expression in the reproductive growth phase

During the reproductive growth phase, the majority of As accumulated in rice remained in the roots at the booting and heading stages, while more As was transported from roots to shoots from heading to milk stages (Fig. 2). Such a translocation of As accumulation in rice plants could be a result of combined regulation by different gene expression in both roots and shoots.

In the roots, the relative expression of *OsABCC1* was still above 1.0 at the booting and heading stages, and the suppression of *OsLsi1* and *OsLsi2* expression at the booting stage was released at the heading stage (Fig. 3), both of which favored As accumulation in roots, resulting in 61.2% of total arsenic accumulated in roots at the heading stage (Fig. 2a). At the same time, the expression of *OsLsi6* in basal stems was promoted at the heading stage (Fig. 4a), which facilitated As translocation to upper nodes and leaves that are connected to the node via transit vascular bundles (TVB). The expression of *OsLsi1*, *OsLsi2* and *OsABCC1* in roots began to be inhibited and decreased over time after the heading stage (Fig. 3). Less As was likely taken up by roots or accumulated in the vacuoles of roots and more As was transferred to the shoots. This result was confirmed by the finding that the TF value increased substantially from the heading to milk stage (Fig. 2b).

When As is transferred to the upper stems, the nodes are vitally important to controlling the arsenic distribution in rice plants [18, 19, 23]. In node III, the expression of *OsABCC1* was substantially higher than that in nodes II and I, particularly at the milk stage (Fig. 4b-d), which could have led to a preferential As accumulation in node III. This result was supported by the finding that the As concentration in node III was higher than that in nodes II and I (*P* > 0.05) (Figure S7). In addition, the expression of *OsABCC1* in node II and I was enhanced at the flowering and heading stages, respectively, indicating that more As accumulated in the vacuoles of the nodes. Our results confirmed that the As concentrations in the nodes were much higher than those in the stems (Figure S7 and Fig. 1b).

In node I, EVBs and DVBs are connected to the top first leaf and the panicle, respectively; therefore, the transfer of elements between EVBs and DVBs determines their relative distribution between the top first leaf and the grains. The expression of *OsLsi6*, *OsLsi2* and *OsLsi3* was simultaneously suppressed at the flowering stage, which can probably attribute to a self-protection of As toxicity to the tissues of node I. Since the cooperation of these three transporters is required for the allocation of As to the panicles via the xylem pathway [22, 23], their simultaneous suppression in node I would reduce As transfer from node I to panicles and increase As transfer to the top first leaf at the flowering stage.

The leaves of rice plants had the highest As concentration and percentage of As contents at the flowering and milk stages relative to that in other organs (Fig. 1 and 2a). This result implies that As transport to leaves via transpirational flow through the xylem vessels still plays an important role in As accumulation in leaves. On the other hand, OsLsi6 in leaves is responsible for the unloading of substrates (including arsenite) out of xylem into the leaf tissues [8, 21]. The stimulated expression of both the *OsLsi6* and *OsABCC1* genes in the bottom and top first leaves suggested that the leaves had a high capacity to accumulate As in their tissues and vacuoles (Fig. 5a and c).

In husks, the relative expression of *OsLsi6* and *OsABCC1* was promoted simultaneously at the flowering stage (Fig. 5). The transient enhancement of *OsLsi6* expression in husks may increase the transport of arsenite through the xylem pathway into the husks. At the same time, the promotion of *OsABCC1* expression could increase the vacuolar segregation of As in the husks. Therefore, upregulation of *OsLsi6* and *OsABCC1* expression at the flowering stage can lead to an increase in As accumulation in husks and a decrease in As distribution to brown rice. Overall, the expression of different genes in roots, nodes, leaves and husks during the reproductive growth phase demonstrated that rice plants have developed various ways to regulate the transport and accumulation of As in plants, which eventually decreases As distribution in brown rice.

During the reproductive phase, regulation of gene expression in different organs of rice is more complicated. On the one hand, the nodes and leaves have upregulated the expression of the *OsLsi6* and *OsABCC1* genes and accumulated the majority of As taken up by rice. This sceanrio can be considered self-regulation of rice in response to arsenic stress. On the other hand, our results also demonstrated that the milk stage is the key period for As distribution in panicles. Therefore, downregulation of *OsLsi6*, *OsLsi2* and *OsLsi3* gene expression in the nodes and upregulation of *OsLsi6* and *OsABCC1* gene expression in the husks are necessary to reduce As transport from nodes to panicles and increase As accumulation in husks.

## Conclusions

Under the stress of arsenite, our results demonstrated that the jointing stage is the key period for the expression of arsenite-transporting genes in roots, and the heading to milky ripe stages are the key period for the expression of arsenite-transporting genes in shoots. The high accumulation of arsenic in roots at the jointing stage was mainly associated with the relatively high expression of *OsLsi2*, *OsLsi1* and *OsABCC1* genes. The substantial increase in arsenic accumulation in shoots during the heading to milk stages was related to the upregulation of *OsLsi3*/*OsLsi6* and *OsABCC1* expression in nodes/leaves and husks as well as the suppression of *OsABCC1* expression in roots. These findings provide useful information on the critical time to apply regulation measures to control the As uptake and transport in rice plants during safe rice production in arsenic-contaminated paddy soil. It should be noted that the results obtained with one rice genotype in this study may not apply to the other rice genotypes; therefore, further investigations are needed to ascertain the expression patters of genes for arsenite uptake and translocation in other rice varieties that are also widely used in the rice production.

## Methods

### Plant materials and growth experiments

The rice (cv. *Oryza sativa* L.) variety Youyou 128, a three-line *indica* hybrid rice cultivar that can accumulate high As in rice grains, was selected [37, 38]. The rice seeds were purchased from Vegetable Research Institute of Guangdong Agricultural Academy. After being surface sterilized in 75% ethyl alcohol and 30% H_2_O_2_, the seeds were thoroughly rinsed with deionized water for 4 h and then placed on a sheet of moist filter paper in the dark at 25 °C. Once germinated, rice seedlings were transplanted into a 2 L plastic pot containing half strength Kimura B solution as a nutrient solution and set as the beginning of the whole growth period (0 week). After 1 week, NaAsO_2_ with a final concentration of 5 μM was added into the nutrient solution in the +As treatment, with a CK treatment that had no As(III) as a control. Three independent biological replicates were set up for each treatment, in which 6 rice plants that grew in the same plastic pot were set as one replicate. The seedlings were grown in an artificial greenhouse at 22–28 °C and 70% relative humidity with a photoperiod of 10:14 h (light/dark). The temperature was then increased gradually to 32 °C, and the photoperiod changed to 12:12 h during the booting and heading stages (12–16 weeks).

The composition of the nutrient solution was as follows: 0.18 mM (NH_4_)_2_SO_4_, 0.27 mM MgSO_4_, 0.09 mM KNO_3_, 0.09 mM KH_2_PO_4_, 0.18 mM Ca(NO_3_)_2_, 0.045 mM K_2_SO_4_, 20 μM NaFe-EDTA, 6.7 μM MnSO_4_, 0.15 μM ZnSO_4_, 0.16 μM CuSO_4_, 9.4 μM H_3_BO_3_, 0.10 μM Na_2_MoO_4_, and 0.10 μM CoSO_4_. The pH of the hydroponic nutrient solution was adjusted to 5.6 with 1.0 M KOH or 1 M HCl, and the nutrient solution was renewed every 3 d during the whole growth period. The nutrient solution was a Si-free formulation with a Si concentration below the detection limit of 0.013 mM to minimize the competitive absorption impact of silicic acid on arsenite uptake by roots [8, 33]. Hydroponics was used in this study because it is more suitable to monitoring the physiological functions of plants (e.g., absorption and translocation of nutrients in plants), while pot or field experiments are more susceptible to the environment [39, 40].

### Sample collection and preparation

The rice plants were collected at seven stages during the whole growth period: (i) seedling stage (3 weeks after transplantation, 3 weeks), (ii) tillering stage (6 weeks), (iii) jointing stage (9 weeks), (iv) booting stage (12 weeks), (v) heading stage (16 weeks), (vi) flowering stage (18 weeks), and (vii) milk stage (20 weeks). At each stage, the harvested plants were washed with distilled water and separated into roots and shoots, with the shoots being subdivided into stems (including leaf sheaths), leaves, panicles, and grains (i.e., husks and brown rice). The roots, stems, leaves, panicles, husks and brown rice were oven dried at 60 °C for 72 h and ground into powder with a mill before As analysis. For the analysis of gene expression, the roots, basal stems, nodes (i.e., node III, II, and I), leaves (i.e., bottom first leaf, top second leaf, and top first leaf), panicles and husks (Figure S8) were frozen and milled into powder in liquid nitrogen, and then stored at − 80 °C before RNA extraction.

### Determination of total as and translocation factor

Approximately 0.2 g of the dried samples was predigested with a 10 mL HNO_3_ and HClO_4_ mixture (87:13, v:v) at room temperature for 8 h and then digested on a graphite digestion apparatus (proD48, Changsha Zerom Instrument and Meter Co., Ltd., Hunan, China) [41]. Then, the digested solution was diluted with 1% HNO_3_ to 50 mL and then filtered with 0.45 μm filter paper. The total As concentration was determined by a hydrogen generation-atomic fluorescence spectrometer (AFS-933, Titan Instruments Co., Ltd., Beijing, China). Certified reference material (GBW10020, citrus leaf flour samples) and a blank were used for quality control. The As recovery from the citrus leaf flour was 111.8 ± 1.9% (*n* = 18).

The translocation from root to shoot was presented as a TF, which was calculated as follows:

1 $$ \mathrm{TF}={\mathrm{C}}_{\mathrm{shoot}}/{\mathrm{C}}_{\mathrm{root}}, $$

where C_shoot_ and C_root_ are As concentrations in the shoots (mg/kg) and roots of rice plants (mg/kg), respectively.

### RNA extraction and reverse transcriptase polymerase chain reaction (RT-PCR)

Total RNA from the plant samples was extracted using Trizol reagent (Invitrogen Corp., CA, USA). Then, first-strand cDNA was synthesized from 1 μg of total RNA using oligo dT (18) primer after removing genomic DNA by a PrimeScript™ RT reagent kit with gDNA eraser (Takara Bio. Inc., Kanagawa, Japan). Relative transcript levels of the genes *OsLsi1*, *OsLsi2*, *OsLsi3*, *OsLsi6* and *OsABCC1* in different parts of rice and *Actin* (internal control) were measured (Table S1). Real-time quantitative RT-PCR was performed in a 10 μL reaction volume containing 2.5 μL of 1:5 diluted cDNA, 500 nM each gene-specific primers and SYBR Premix Ex Taq (Takara Bio. Inc., Kanagawa, Japan) using CFX384 Real-Time System (CFX384 touch, Bio-Rad Laboratories Inc., CA, USA). Real-time quantitative PCR was performed using the following protocol: (94 °C/2 min) × 1; (94 °C/30 s)/(58 °C/30 s)/(72 °C/30 s) × 45; and (72 °C/5 min) × 1. The specific primer sequences for the genes *OsLsi1*, *OsLsi2*, *OsLsi3*, *OsLsi6*, *OsABCC1* and *Actin* are shown in Table S2. The target gene expression was normalized based on *Actin* in the CK treatment by the 2^-ΔΔCt^ method as follows [42]:

2 $$ {\Delta  \mathrm{C}}_{\mathrm{t}}={\mathrm{C}}_{\left(\mathrm{t},\mathrm{target}\ \mathrm{gene}\right)}-{\mathrm{C}}_{\left(\mathrm{t},\mathrm{internal}\ \mathrm{control}\ \mathrm{gene}\right)} $$

3 $$ {\Delta  \Delta  \mathrm{C}}_{\mathrm{t}}={\Delta  \mathrm{C}}_{\left(\mathrm{t},+\mathrm{As}\right)}-{\Delta  \mathrm{C}}_{\left(\mathrm{t},\mathrm{CK}\right)} $$

4 $$ \mathrm{Relative}\ \mathrm{expression}\ \mathrm{level}={2}^{-\Delta  \Delta  \mathrm{Ct}} $$

C_(t, target gene)_ and C_(t, internal control gene)_ are the threshold cycles of the target gene and *Actin* amplification, respectively. ΔC_(t, +As)_ and ΔC_(t, CK)_ are equal to the difference in threshold cycles for the target and internal control genes in the +As and CK treatments, respectively.

### Statistical analysis

All statistical analyses were performed with SPSS 19.0 software (SPSS Inc., IL, USA). The significance of the difference among the growth stages was analysed by one-way ANOVA. A one-sample *t*-test was used to detect significant differences. The graphs were created by Origin 8.0 (OriginLab, Mass, USA).

## Supplementary information

**Additional file 1 Figure S1.** Biomass of different parts of the rice plants during the whole growth period. **Figure S2.** Total arsenic contents in different parts of the rice plants during the whole growth period. **Figure S3.** Correlations between the relative expression of *OsLsi1* and *OsABCC1* (a) and between the relative expression of *OsLsi2* and *OsABCC1* in rice roots (b) during the whole growth period of rice plants. **Figure S4.** Relative expression of *OsPCS1* gene in roots in the +As treatment during the whole growth period. **Figure S5.** Relative expression of *OsPCS1* gene in (a) basal stem, (b) node III, (c) node II, and (d) node I of rice in the +As treatment during the whole growth period. **Figure S6.** Relative expression of *OsPCS1* gene in (a) bottom first leaf, (b) top second leaf, (c) top first leaf, and (d) husk in the +As treatment during whole growth period. **Figure S7.** Total As concentration in nodes at the milk stage in the +As treatment. **Figure S8.** Schematic diagram of rice samples harvested. **Table S1.** Target genes in different tissues were determined in the experiment. **Table S2.** Specific primer sequences of the genes in the experiment.

**Additional file 2.** Data of the means with standard errors for three replicates in Figs. 1, 2, 3, 4 and 5, S1-S2, and S4-S7.

## Data Availability

All data generated or analysed during this study are included in this published article and its supplementary information files or are available from the corresponding author on request.

## Abbreviations

As(III): Arsenite
EVBs: Enlarged vascular bundles
DVBs: Diffuse vascular bundles
PCs: Phytochelatins
RT-PCR: Reverse transcriptase polymerase chain reaction
TF: Translocation factor
TVB: Transit vascular bundles
