# Supplementary material for: Dynamics of gene expression associated with arsenic uptake and transport in rice during the whole growth period
Source: BMC Plant Biol. 2020 Mar 31;20:133. doi: 10.1186/s12870-020-02343-1 (PMC7106585; doi:10.1186/s12870-020-02343-1)
Supplement: Supplementary file 1 — Additional file 1 Figure S1. Biomass of different parts of the rice plants during the whole growth period. Figure S2. Total arsenic contents in different parts of the rice plants during the whole growth period. Figure S3. Correlations between the relative expression of OsLsi1 and OsABCC1 (a) and between the relative expression of OsLsi2 and OsABCC1 in rice roots (b) during the whole growth period of rice plants. Figure S4. Relative expression of OsPCS1 gene in roots in the +As treatment during the whole growth period. Figure S5. Relative expression of OsPCS1 gene in (a) basal stem, (b) node III, (c) node II, and (d) node I of rice in the +As treatment during the whole growth period. Figure S6. Relative expression of OsPCS1 gene in (a) bottom first leaf, (b) top second leaf, (c) top first leaf, and (d) husk in the +As treatment during whole growth period. Figure S7. Total As concentration in nodes at the milk stage in the +As treatment. Figure S8. Schematic diagram of rice samples harvested. Table S1. Target genes in different tissues were determined in the experiment. Table S2. Specific primer sequences of the genes in the experiment. [file 12870_2020_2343_MOESM1_ESM.docx]

**Supplementary information for**

**Dynamics of gene expression associated with arsenic uptake and transport in rice during the whole growth period**

Dandan Pan^1,2,3,6^, Jicai Yi^4^, Fangbai Li^2^, Xiaomin Li^1,6,*^, Chuanping Liu^2^, Weijian Wu^2^ and Tingting Tao^2,5^

^1^*SCNU Environmental Research Institute, Guangdong Provincial Key Laboratory of Chemical Pollution and Environmental Safety & MOE Key Laboratory of Theoretical Chemistry of Environment, South China Normal University, Guangzhou 510006, China*

^2^*Guangdong Institute of Eco-Environmental Science & Technology, Guangdong Key Laboratory of Integrated Agro-environmental Pollution Control and Management, Guangzhou 510650, China*

^3^*College of Natural Resources and Environment, South China Agricultural University, Guangzhou 510642, China*

^4^*College of Life Sciences, South China Agricultural University, Guangzhou 510642, China*

^5^*School of Food Science and Engineering, Foshan University, Foshan 528000, China*

^6^*School of Environment, South China Normal University, Guangzhou 510006, China*

*Corresponding author

Mailing address: No. 378 Waihuan Xi Road, Guangzhou 510006, China

Phone: +86-20-39311270

Email: [xiaomin.li@m.scnu.edu.cn](mailto:xiaomin.li@m.scnu.edu.cn) (Xiaomin Li)

**

**

**Supplementary Figure S1.** Biomass of different parts of the rice plants during the whole growth period. (a) root, (b) stem, (c) leaf, and (d) panicle and grain. Data are presented as the mean ± SE (*n* = 3). Significant differences between the CK and +As treatments are indicated by * at a *P* < 0.05 level.

**

**

**Supplementary Figure S2.** Total arsenic contents in different parts of the rice plants during the whole growth period. (a) root, (b) stem, (c) leaf, (d) husk and brown rice. Plants were grown under hydroponic conditions with 5 μM NaAsO_2_ (+As treatment) or without it (the control, CK). Data are presented as the mean ± SE (*n* = 3). Different letters with colors corresponding to their respective lines indicate significant differences (*P* < 0.05) among values at different time intervals in the +As treatment.

**
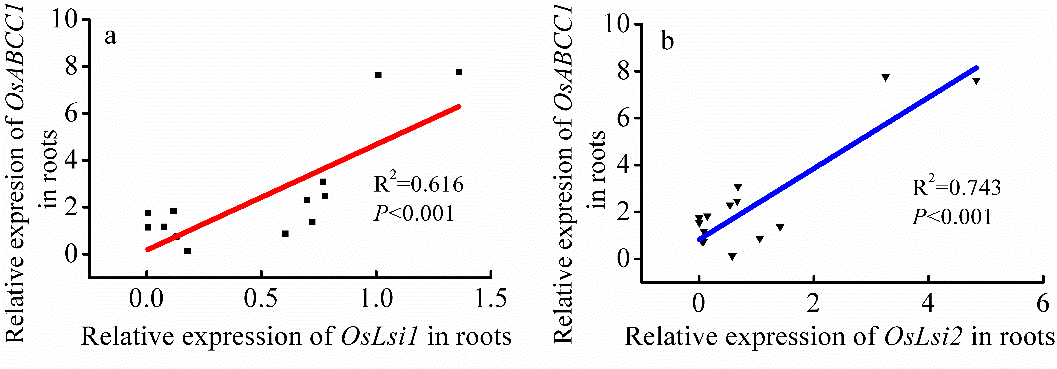
**

**Supplementary Figure S3.** Correlations between the relative expression of *OsLsi1* and *OsABCC1* (a) and between the relative expression of *OsLsi2* and *OsABCC1* in rice roots (b) during the whole growth period of the +As treatment.

**

**

**Supplementary Figure S4.** Relative expression of *OsPCS1* gene in the roots of rice in the +As treatment relative to those in the control treatment during the whole growth period. Data are presented as the mean ± SE (*n* = 3). Lines labeled by * with colors corresponding to their respective lines are significantly different at a *P* < 0.05 level in comparison to the control treatment. Those of *OsABCC1* gene are from Fig. 3 and for comparison only herein.

**

**

**Supplementary Figure S5.** Relative expression of *OsPCS1* gene in (a) basal stem, (b) node III, (c) node II, and (d) node I of rice in the +As treatment relative to those in the control treatment during the whole growth period. Data are presented as the mean ± SE (*n* = 3). Lines labeled by * with colors corresponding to their respective lines are significantly different at a *P* < 0.05 level in comparison to the control treatment. Those of *OsABCC1* gene are from Fig. 4 and for comparison only herein.

**

**

**Supplementary** **Figure S6.** Relative expression of *OsPCS1* gene in (a) bottom first leaf, (b) top second leaf, (c) top first leaf, and (d) husk of rice in the +As treatment relative to those in the control treatment during the whole growth period. Data are presented as the mean ± SE (*n* = 3). Lines labeled by * with colors corresponding to their respective lines are significantly different at a *P* < 0.05 level in comparison to the control treatment. Those of *OsABCC1* gene are from Fig. 5 and for comparison only herein.

**
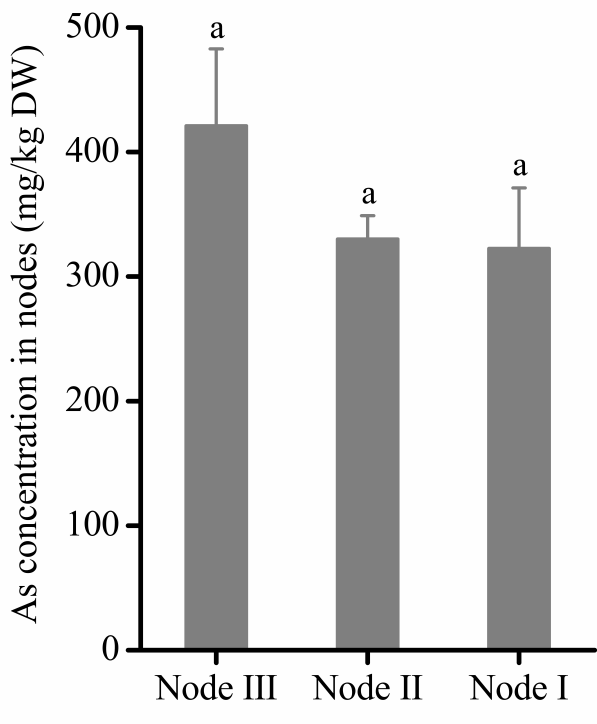
**

**Supplementary Figure S7.** Total As concentration in the nodes at the milk stage in the +As treatment. Data are presented as the mean ± SE (*n* = 3). The same letter indicates no significant difference (*P* > 0.05) among the nodes.


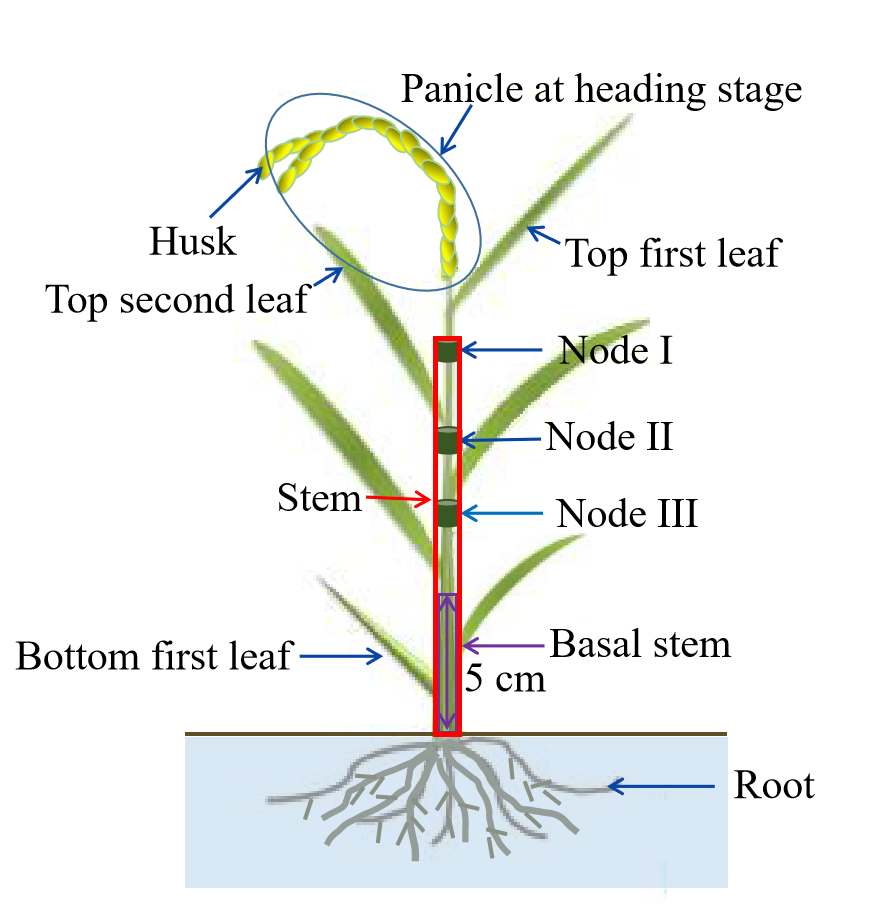


**Supplementary Figure S8.** Schematic diagram of rice samples harvested.

**Table S1.** Target genes in different tissues were determined in the experiment.

| Tissues | | Genes | | | | |
| --- | --- | --- | --- | --- | --- | --- |
| Shoot | Husk |  |  |  | *OsLsi6* | *OsABCC1* |
|  | Top first leaf |  |  |  | *OsLsi6* | *OsABCC1* |
|  | Top second leaf |  |  |  | *OsLsi6* | *OsABCC1* |
|  | Node I |  | *OsLsi2* | *OsLsi3* | *OsLsi6* | *OsABCC1* |
|  | Node II |  | *OsLsi2* | *OsLsi3* | *OsLsi6* | *OsABCC1* |
|  | Node III |  | *OsLsi2* | *OsLsi3* | *OsLsi6* | *OsABCC1* |
|  | Bottom first leaf |  |  |  | *OsLsi6* | *OsABCC1* |
|  | Basal stem |  |  |  | *OsLsi6* | *OsABCC1* |
| Root | | *OsLsi1* | *OsLsi2* |  |  | *OsABCC1* |

**Table S2.** Specific primer sequences of the genes in the experiment [1-4].

| Genes | Forward primers | Reverse primers |
| --- | --- | --- |
| *Actin* | 5`-GACTCTGGTGATGGTGTCAGC-3` | 5`-GGCTGGAAGAGGACCTCAGG-3` |
| *OsLsi1* | 5`-CGGTGGATGTGATCGGAACCA-3` | 5`-CGTCGAAC TTGTTGCTCGCCA-3` |
| *OsLsi2* | 5`-ATCTGGGACTTCATGGCCC-3` | 5`-ACGTTTGATGCGAGGTTGG-3` |
| *OsLsi3* | 5`-CTGTATCCCTGTTGCCAGCTG-3` | 5`-TAATCCGGCATGCGTACTTG-3` |
| *OsLsi6* | 5`-GAGTTCGACAACGTCTAATCGC-3` | 5`-AGTACACGGTACATGTATACACG-3` |
| *OsABCC1* | 5`-AACAGTGGCTTATGTTCCTCAAG-3` | 5`-AACTCCTCTTTCTCCAATCTCTG-3` |

**References**

1. Ma JF, Tamai K, Yamaji N, Mitani N, Konishi S, Katsuhara M, Ishiguro M, Murata Y, Yano M. A silicon transporter in rice. Nature 2006; doi:10.1038/nature04590.
2. Song WY, Yamaki T, Yamaji N, Ko D, Jung KH, Fujii-Kashino M, An G, Martinoia E, Lee Y, Ma JF. A rice ABC transporter, OsABCC1, reduces arsenic accumulation in the grain. Proc. Natl. Acad. Sci. USA. 2014; doi:10.1073/pnas.1414968111.
3. Yamaji N, Sakurai G, Mitani-Ueno N, Ma JF. Orchestration of three transporters and distinct vascular structures in node for intervascular transfer of silicon in rice. Proc. Natl. Acad. Sci. USA. 2015; doi:10.1073/nas.1508987112.
4. Yamaji N, Ma JF. A transporter at the node responsible for intervascular transfer of silicon in rice. Plant Cell 2009; doi:10.1105/tpc.109.069831.
